# Alexithymia and anxiety in female chronic pain patients

**DOI:** 10.1186/1744-859X-5-13

**Published:** 2006-08-15

**Authors:** Feryal Cam Celikel, Omer Saatcioglu

**Affiliations:** 1 Gaziosmanpasa University School of Medicine 60100 Tokat, Turkey; 2 Bakirkoy Research Hospital for Psychiatric and Neurological Diseases, Alcohol and Drug Treatment and Research Center (Amatem), Istanbul, Turkey

## Abstract

**Objectives:**

Alexithymia is highly prevalent among chronic pain patients. Pain is a remarkable cause for high levels of chronic anxiety. The purpose of this study was to investigate the prevalence of alexithymia and to determine anxiety levels among DSM-IV somatoform pain disorder (chronic pain) female patients and to examine the relationship between alexithymia and the self-reporting of pain.

**Methods:**

Thirty adult females (mean age: 34,63 ± 10,62 years), who applied to the outpatient psychiatry clinic at a public hospital with the diagnosis of chronic pain disorder (DSM-IV), were included in the study. Thirty seven healthy females (mean age: 34,46 ± 7,43 years), who matched for sociodemographic features with the patient group, consisted the control group. A sociodemographic data form, 26-item Toronto Alexithymia Scale (TAS-26), Spielberger Trait Anxiety Inventory (STAI) were administered to each subject and information was obtained on several aspects of the patients' pain, including intensity (measured by VAS), and duration.

**Results:**

Chronic pain patients were found significantly more alexithymic than controls. There was a positive correlation between TAS-26 scores and the duration of pain. The alexithymic and nonalexithymic group did not differ in their perception of pain. Neither positive correlation nor significant difference was found between alexithymia and trait anxiety in pain patients.

**Discussion:**

Alexithymia may be important in addressing the diversity of subjective factors involved in pain. The conceptualization of alexithymia as a personality trait as well as a secondary state reaction is underlined by our data.

## Background

The original definition of alexithymia is the inability to identify and use verbal language to describe feelings [1,2]. Alexithymia has been associated with a variety of psychiatric disorders as well as physical illness [3-10]. As a measure, Toronto Alexithymia Scale was significantly correlated with the measures of the tendency to experience and report physical signs and symptoms [11].

Several studies have found a high prevalence of alexithymia in pain patients. Chronic pain patients frequently exhibit many of the core features of alexithymia, such as problems in identifying and describing subjective feelings, impoverished imaginative abilities, and excessive preoccupation with physical symptoms and external events. Although several studies have found a high prevalence of alexithymia in pain patients, the way alexithymia may possibly influence pain experience is still unclear [12,13].

DSM-IV-TR defines pain disorder as the presence of pain that is "the predominant focus of clinical attention" [14]. In chronic pain disorder, patients complain of chronic pain, for which no physical etiology could be found or the underlying disorder is insufficient in explaining the symptoms. The pain causes clinically significant distress or impairment in social, occupational, or other important areas of functioning. Psychological factors are judged to have an important role in the onset, severity, exacerbation, or maintenance of the pain [15].

The alexithymic person's difficulty in identifying and describing feelings may increase symptom reporting by several mechanisms. Consequently, due to the difficulty to experience and express emotions, alexithymia has been linked with somatosensory amplification, which is the tendency to focus on benign somatic sensations. Alexithymic subjects are considered to focus on somatic manifestations of emotional arousal, resulting in misinterpretation of somatic sensations as signs of physical illness [12,13,16]. Accordingly, previous studies have found evidence of an association between alexithymia and the development of functional somatic symptoms, as seen in patients with somatoform disorders. On the other hand, alexithymia may also occur as a secondary state reaction in response to severe and chronic medical illness [17-21].

Based on previous findings, these factors are worth receiving more attention in terms of clinical research. The purpose of the present study was to investigate the prevalence of alexithymia among DSM-IV somatoform pain disorder (chronic pain) female patients and to examine the relationship between alexithymia and the self-reporting of pain in this group of patients. Besides, the study searched for the anxiety levels of chronic pain patients with or without alexithymia.

## Materials and methods

### Sample

The sample consisted of 30 females who applied to the outpatient psychiatry clinic at a public hospital and who met DSM-IV diagnostic criteria for chronic pain disorder. Patients with concomitant psychiatric disorders, such as major depression, anxiety disorders and somatoform disorders other than pain disorder were excluded.

Patients either directly applied to the psychiatry clinic themselves or were referred for psychiatric assessment from another outpatient clinic, mainly physical medicine and rehabilitation. After complete description of the study, written informed consent was obtained from each subject.

The control group was 37 healthy females, who matched for age, and education with the subjects. All subjects participated voluntarily in the study and gave consent after the procedure had been fully explained to them.

The mean age of the patients and the healthy controls was 34,63 ± 10,62 (range: 16–62) and 34,46 ± 7,43 (range: 22–57) years and the educational level was 6,13 ± 3,03 (range: 5–11) and 6,59 ± 2,9 (range: 5–14) years, respectively. There were no significant differences between the two groups with respect to age (*t *= 0,79, *df *= 65, *P *> 0,05), educational level (*t *= 1,02, *df *= 65, *P *> 0,05), and marital status (*x*^2 ^= 0,51, *df *= 1, *P *> 0,05).

### Measures

A detailed sociodemographic data form was used for all subjects. All participants were applied Structured Clinical Interview for DSM-IV (SCID-I) [22], Turkish version [23]. Regarding the pain assessment, information was first obtained on several aspects of the patients' pain, such as intensity, and duration. Pain intensity was measured by Visual Analogue Scale (VAS), using a horizontal 10-cm line with the statement 'no pain at all' at the extreme left-hand end and 'the worst possible pain' or 'unbearable' at the right-hand extreme. VAS is scored by measuring the distance from the end of the scale indicating absence of pain (or no distress or no pain relief) to the place marked by the patient [24].

The psychometric scales used in the study were the 26-item Toronto Alexithymia Scale (TAS-26] and the Trait Anxiety Inventory (STAI), which were both validated in Turkish population studies [25-28]. TAS is a psychometrically well validated and reliable instrument in the assessment of alexithymia. TAS has been validated in Turkish studies as a true or false scale. Twenty-six items are scored either as 1 or 0 and the higher scores indicate higher degrees of alexithymia. TAS has an interval consistency of 0.65 [Kuder-Richardson) and test-retest reliability is r = 0.71, p < 0.01 in Turkish reliability and validity study. The sample was divided into nonalexithymic and alexityhmic groups, with the recommended cut-off score of 11 [27]. Spielberger Trait Anxiety Inventory (STAI) is one of the two sections of the Spielberger Anxiety Inventory (the other, measuring state anxiety). 'Trait anxiety' has been defined as anxiety proneness, that is, the tendency to respond to situations perceived as threatening with elevations in the intensity of state anxiety [26].

### Statistical analysis

In order to determine the relative importance of a number of factors in pain disorders, we used both correlation analyses. The alexithymic and nonalexithymic groups were compared using the independent sample t-tests on scores of psychological tests. The statistical procedure, which was carried out by a SPSS package program for Windows using Chi-square, Fisher's exact test, two tailed t test and Pearson correlation coefficients, was also used to determine group differences (alexithymics versus nonalexithymics) in sociodemographic variables and various aspects of pain.

## Results

In the chronic pain group, 56.7% of patients (n = 17) had a score greater than 11 on the TAS-26, and were considered alexithymic. The mean TAS-26 score of the alexithymic group (n = 17) was 17.88 ± 3.43 and the nonalexithymic group (n = 13) was 8.39 ± 2.02. Age (t = 1,38, df = 28, p > 0,18), education (t = -0,21, df = 28, p > 0,16) and marital status (x^2 ^= 0,27, df = 1, p > 0,87) were not associated with alexithymia (Table 1).

**Table 1:** Comparison of Sociodemographic Data of the Chronic Pain Patients and Control Subjects

	Female Pain Patients			Control Subjects		
	Alexithymics (n = 17)	Nonalexithymics (n = 13)	P	Alexithymics (n = 9)	Nonalexithymics (n = 28)	P

Age, median ± SD (yr)	36,94 ± 11,69	31,62 ± 8,55	NS	36,78 ± 6,83	33,71 ± 7,57	NS
Education, median ± SD (yr)	6,24 ± 3,63	6,0 ± 2,12	NS	5,67 ± 2	6,89 ± 3,10	NS
Marital status, n (%)						
Single	3	2	NS	3	8	NS
Married	14	11		6	20	

Abbreviation: NS, not significant

In the control group, 24,3% of patients (n = 9) were alexithymic according to TAS-26. The mean TAS-26 score of the alexithymic group (n = 9) was 13,82 ± 1,93 and the nonalexithymic group (n = 28) was 10,33 ± 0,86. Alexithymia was not associated with age (t = -1,08, df = 35, p > 0,29), educational level (t = 1,1, df = 35, p > 0,28), or marital status (x^2 ^= 0,74, df = 1, p > 0,79) or anxiety levels in the control subjects (Table 1).

The duration and severity of pain, TAS-26 scores, and STAI scores of the female pain patients are shown in Table 2. Comparison of the alexithymics with nonalexithymics on either the severity of pain or pain duration showed no statistical significance (t = 0,64, df = 28, p > 0,52, t = 2,05, df = 28, p > 0,05, respectively).

**Table 2:** Duration of Pain, Severity of Pain, TAS-26 Scores, and STAI Scores of the Female Pain Sample

	FEMALE PAIN GROUP				
	Alexithymics (n = 17)	Nonalexithymics (n = 13)	*t*	*df*	*P*

Duration of pain, median ± SD (yr)	7,44 ± 6,82	3,31 ± 2,79	2,05	28	NS
Severity of pain, median ± SD (yr)	7,12 ± 2,98	6,46 ± 2,40	0,64	28	NS
TAS-26 score	17,88 ± 3,43	13,85 ± 3,99	2,98	28	0,006*
STAI score	47,88 ± 9,89	47,69 ± 5,36	0,06	28	NS

Abbreviation: NS, not significant

*p < 0.05, statistically significant

TAS-26 score and duration of pain were found positively correlated (r = 0,50, n = 30, p > 0,005). STAI (trait) scores of the alexithymics in the pain group did not significantly differ from the nonlalexithymics (t = 0,06, df = 28, p > 0,95) and besides, TAS-26 and STAI scores were not correlated (r = 0,06, p > 0,72).

In summary, there are three points to be emphasized. First, chronic pain patients were found significantly more alexithymic than controls (56,7% to 24,3%). Second, a positive correlation was observed between TAS-26 scores and duration of pain. Third, neither positive correlation nor significant difference was found between alexithymia and trait anxiety in pain patients.

## Discussion

The results of the present study suggest that patients with chronic pain disorder are more alexithymic than individuals with no pain. This finding is consistent with results obtained with earlier measures of alexithymia [11-13]. Although they may share common clinical features, alexithymia and somatoform pain are independent constructs. Alexithymia may be a consequence to the effects of severe physical symptoms, such as a reduced quality of life and limitations in daily activities. Besides, alexithymia may be conceptualized as a personality trait as well as a secondary state reaction [2,3,15-17]. In this study, the question investigated was whether alexithymia has any correlation with the duration or severity of the pain itself.

There were no significant differences between alexithymic and nonalexitymic patients on self reports of current pain severity. This is in accordance with Cox's study [1994] in which it was further pointed out that alexithymic patients were found to use significantly more verbal descriptors of pain compared to nonalexithymic patients [13]. In our study, pain intensity was only evaluated by using VAS. One problem in trying to measure the intensity of pain is the lack of an objective way. Pain is a subjective experience and each patient may communicate in a different way, verbally or nonverbally [29]. Patients in this sample were sufferers of chronic pain, who had already chosen an approved way of expressing their distress. Since this is true regardless of alexithymia, alexithymic groups and nonalexithymic groups in this sample showed no difference on pain severity.

The positive correlation between alexithymia and the duration of pain in this sample supports the assumption of a two-way hypothesis. It is often assumed that pain can be caused by alexithymic personality traits and also that severe and chronic pain may cause emotional change. One of the limitations of this study is that because of the cross-sectional design, we are unable to draw conclusions about the direction of causality between alexithymia and pain. The duration of the patients' pain could approximately be determined, yet the preexisting level of alexithymia was not known. In the usual absence of internal stimuli, alexithymic person may be expected to maintain an external focus of attention, such as pain. Symptom chronicity may force the alexithymic person to attent to and amplify this somatic sensation.

Difficulties in the ability to identify and differentiate emotions and somatic experiences are core features of the alexithymic construct. Therefore, alexithymic patients might be expected to differ from nonalexithymic ones in their anxiety levels. Yet, in our pain group alexithymic patients showed no significant difference from the nonalexithymics on trait anxiety. Besides, alexithymia and anxiety were not correlated at all. The reasons may be lying in the specific characteristics of this patient group itself.

The study included patients suffering from chronic symptoms; with an average of 7,44 ± 6,82 years of pain in the alexithymic and 3,31 ± 2,79 years in the nonalexithymic groups. Persistency of any physical symptom may bring along alexithymia as a coping strategy. In their paper, Crook and Tunks (1988) examined the types of coping strategies used by persistent pain sufferers and addressed to the importance to alter their attitudes and behavior that tend toward catastrophizing, avoidance and withdrawal, rather than simply concentrate on trying to teach them techniques for 'coping with stress' to help persistent pain sufferers [30]. Sufferers of chronic symptoms in this sample were members of a subgroup who have been seeking medical care for a long time and besides given the chance of being referred to a psychiatrist. Therefore, alexithymic or not, their anxiety might have induced unique coping strategies and illness behavior.

Alexithymia may be important in addressing the diversity of subjective factors involved in pain [31]. It is not known whether it should be addressed in the treatment of pain patients, but a high level of alexithymia may effect the nature of assessment. In summary, the conceptualization of alexithymia as a personality trait as well as a secondary state reaction is underlined by our data. However, regarding the cross-sectional design of this study, only limited conclusions can be drawn about the nature of the causal relationship between alexithymia and chronic pain. Therefore, future longitudinal studies assessing the cause of alexithymic characteristics are required to fully elucidate the concepts of primary and secondary alexithymia.
